# Supplementary material for: Reconciling Mining with the Conservation of Cave Biodiversity: A Quantitative Baseline to Help Establish Conservation Priorities
Source: PLoS One. 2016 Dec 20;11(12):e0168348. doi: 10.1371/journal.pone.0168348 (PMC5173368; doi:10.1371/journal.pone.0168348)
Supplement: S1 Dataset — (ZIP) [file pone.0168348.s002.zip › Taxa/Serra Norte/SN_2013/N4E/Lista N4E-27.pdf]

CAVIDADE N4E-0027

Classe	Ordem	Fam/Outros	Gên/Outros	Espécie	S-U	U-S	Zon.
Arachnida	Acari	Podocinidae	*Podocinum*	sp.1	X		E
Arachnida	Araneae	Pholcidae	*Leptopholcus*	sp.1	X		E
Arachnida	Araneae	Pholcidae	*Mesabolivar*	sp.		X	E
Arachnida	Araneae	Salticidae		sp.		X	E
Arachnida	Araneae	Scytodidae	*Scytodes*	*eleonorae*		1	E
Arachnida	Araneae	Theridiosomatidae	*Plato*	sp.		X	E
Arachnida	Opiliones	Manaosbiidae	*Saramacia*	sp.1	1		E
Arachnida	Opiliones	Stygnidae	*Stygnus*	*luteus*		X	E
Chilopoda	Scutigeromorpha	Pselliodidae	*Sphendononema*	*guildingii*		X	E
Entognatha	Collembola	Isotomidae		sp.1	X		E
Entognatha	Collembola	Paronellidae		sp.1	X		E
Entognatha	Collembola			sp.		X	E
Insecta	Diptera			sp.		X	E
Insecta	Hymenoptera	Formicidae	*Paratrechina*	sp.1	X		E
Insecta	Lepidoptera	Tineoidea		sp.1	X	X	E
Insecta	Orthoptera	Phalangopsidae	*Paraclodes*	sp.	2		E
Insecta	Orthoptera	Phalangopsidae	*Phalangopsis*	sp.		X	E
Insecta	Psocoptera	Epipsocidae	*Epipsocus*	sp.2	X		E
Insecta	Thysanura	Nicoletiidae	Nicoletiinae	sp.1		X	E
Gastropoda	Pulmonata	Subulinidae	*Leptinaria*	sp.		X	E
Gastropoda	Pulmonata	Systrophiidae	*Happia*	sp.		X	E
Amphibia	Anura			sp.	1		E
Mammalia	Chiroptera	Emballonuridae	*Peropteryx*	*kappleri*	2		E
Mammalia	Chiroptera			sp.		1	E
